# Integration analysis of tumor metagenome and peripheral immunity data of diffuse large-B cell lymphoma

**DOI:** 10.3389/fimmu.2023.1146861

**Published:** 2023-05-09

**Authors:** Yu Zhang, Shuiyun Han, Xibing Xiao, Lu Zheng, Yingying Chen, Zhijian Zhang, Xinfang Gao, Shujuan Zhou, Kang Yu, Li Huang, Jiaping Fu, Yongwei Hong, Jinhong Jiang, Wenbin Qian, Haiyan Yang, Jianping Shen

**Affiliations:** ^1^ Department of Hematology, First Affiliated Hospital of Zhejiang Chinese Medical University, Hangzhou, China; ^2^ Department of Lymphoma, Cancer Hospital of University of Chinese Academy of Sciences, Hangzhou, China; ^3^ Department of Hematology, The Second Affiliated Hospital of Zhejiang University School of Medicine, Hangzhou, China; ^4^ Department of Hematology, Lishui People’s Hospital, Lishui, China; ^5^ Department of Hematology, Ningbo Yinzhou No.2 Hospital, Ningbo, China; ^6^ Department of Hematology, Shaoxing People’s Hospital, Shaoxing, China; ^7^ Department of Hematology, Jinhua People’s Hospital, Jinhua, China; ^8^ Department of Hematology, The First Hospital Affiliated to Wenzhou Medical University, Weizhou, China

**Keywords:** diffuse large B-cell lymphoma, gut microbiota, National Comprehensive Center Network-International Prognostic Index, metagenomic sequencing, immunity

## Abstract

**Background/purpose:**

It has been demonstrated that gut microbes are closely associated with the pathogenesis of lymphoma, but the gut microbe landscape and its association with immune cells in diffuse large B-cell lymphoma (DLBCL) remain largely unknown. In this study, we explored the associations between gut microbiota, clinical features and peripheral blood immune cell subtypes in DLBCL.

**Method:**

A total of 87 newly diagnosed DLBCL adults were enrolled in this study. The peripheral blood samples were collected from all patients and then submitted to immune cell subtyping using full-spectral flow cytometry. Metagenomic sequencing was applied to assess the microbiota landscape of 69 of 87 newly diagnosed DLBCL patients. The microbiotas and peripheral blood immune cell subsets with significant differences between different National Comprehensive Center Network-International Prognostic Indexes (NCCN-IPIs) (low-risk, low-intermediate-risk, intermediate-high-risk, high-risk) groups were screened.

**Results:**

A total of 10 bacterial phyla, 31 orders and 455 bacteria species were identified in 69 patients with newly diagnosed DLBCL. The abundances of 6 bacteria, including *Blautia* sp.*CAG 257*, *Actinomyces* sp.*S6 Spd3*, *Streptococcus parasanguinis*, *Bacteroides salyersiae*, *Enterococcus faecalls* and *Streptococcus salivarius* were significantly different between the low-risk, low-intermediate-risk, intermediate-high-risk and high-risk groups, among which *Streptococcus parasanguinis* and *Streptococcus salivarius* were markedly accumulated in the high-risk group. The different bacteria species were mostly enriched in the Pyridoxal 5’-phosphate biosynthesis I pathway. In addition, we found that 2 of the 6 bacteria showed close associations with the different immune cell subtypes which were also identified from different NCCN-IPIs. In detail, the abundance of *Bacteroides salyersiae* was negatively correlated with Treg cells, CD38+ nonrescue exhausted T cells, nature killer 3 cells and CD38+CD8+ effector memory T cells, while the abundance of *Streptococcus parasanguinis* was negatively correlated with HLA-DR+ NK cells, CD4+ Treg cells, HLA-DR+ NKT cells and HLA-DR+CD94+CD159c+ NKT cells.

**Conclusion:**

This study first reveals the gut microbiota landscape of patients with newly diagnosed DLBCL and highlights the association between the gut microbiota and immunity, which may provide a new idea for the prognosis assessment and treatment of DLBCL.

## 1 Introduction

Lymphoma, a malignant tumor originating from the lymphohematopoietic system, is one of the most common cancer in both men and women (1). Diffuse large B-cell lymphoma (DLBCL) is the most common subtype of lymphoma worldwide, accounting for 40% of non-Hodgkin lymphomas (2). Despite R-CHOP regimen (rituximab, cyclophosphamide, doxorubicin, vincristine, and prednisone) significantly improves the complete remission rate of DLBCL, one-third patients still do not benefit from it and have a poor prognosis (3). It is urgent to deeply reveal the mechanisms underlying the pathogenesis of DLBCL and develop potent therapeutic strategy.

Microbiome is becoming crucial in maintaining the balance between human health and diseases. Harmful gut microbiotas trigger fatigue, dry skin, headaches, vomiting and other physical discomforts, or accelerate the aging of the intestinal wall, produce carcinogens and eventually lead to gastrointestinal cancers (4, 5). Also, intestinal microecology plays an important role in the development and progression of hematological malignancies (6). Gao et al. (7) found that the dominant intestinal microflora constitutions in children with acute lymphoblastic leukemia were significantly different from healthy controls, with a poorer gut microbial diversity. Cozen et al. (8) using the 16S ribosomal RNA sequencing revealed a lower microbial diversity in patients with Hodgkin’s lymphoma as compared to healthy individuals. Additionally, the gut microbiotas play an indispensable role in the pathogenesis of DLBCL (9). In detail, higher abundance of *Proteobacteria* was found to be associated with lower immunity and poor survival of DLBCL patients (9). Moreover, the gut microbial components were associated with the response to checkpoint blockade therapies (CBT) and prognosis of cancers (10). For example, the alpha diversity and relative abundance of bacteria of the *Ruminococcaceae* family were significantly increased in the responders to anti-PD-1 immunotherapy as compared with the nonresponders in melanoma (11). Perturbations of the microbiota composition (*B. fragilis* and/or *B. thetaiotaomicron* and *Burkholderiales*) improved the efficacy of CTLA-4 blockade through promoting T cell-dependent intestinal epithelial cell death (12). However, the profiles and clinical significances of gut microbiota in DLBCL have not been fully understood.

Peripheral immune system, as the first immune barrier of human body, plays an important role in the diagnosis and prognosis of DLBCL (13, 14). For instance, the absolute peripheral monocyte count at diagnosis is an independent risk factor to predict the central nervous system relapse in patients with DLBCL (15). The higher proportion of helper cells is associated with shorter survival in DLBCL patients, especially in younger patients (14). Noticeably, gastrointestinal tract provides a critical interface where crosstalk between the enormous number of microorganisms and the host immune system takes place, allowing the gut microbiome modulates the host immune system both locally and systemically (16). Exploration of the relationship between gut microbial and immune system may provide a new idea for the treatment of DLBCL.

International Prognostic Index (IPI), revised IPI (R-IPI), and National Comprehensive Cancer Network IPI (NCCN-IPI) are the three scoring systems used to predict the prognosis of DLBCL. Among them, NCCN-IPI outperforms the IPI and R-IPI systems (17). To disclose the relationship between gut microbiota and prognosis, we investigated the association between intestinal bacteria and the NCCN-IPI in DLBCL patients. In addition, we studied the associations between intestinal bacteria and immune cell subtypes. This study may provide novel biomarkers used for DLBCL prognostic prediction and new ideas for treatment strategy.

## 2 Materials and methods

### 2.1 Sample collection and preparation

A total of 87 newly diagnosed DLBCL patients were recruited from eight hospitals of Zhejiang Province, including the First Affiliated Hospital of Zhejiang Chinese Medical University (n=14), the Second Affiliated Hospital of Zhejiang University School of Medicine (n=16), Cancer Hospital of the University of Chinese Academy of Sciences (n=31), the First Affiliated Hospital of Wenzhou Medical University (n=3), Lishui People’s Hospital (n=6), Shaoxing People’s Hospital (n=7), Jinhua People’s Hospital (n=3) and Ningbo Yinzhou No.2 Hospital (n=7) between 2020 and 2021 (**Table 1**). The diagnosis of DLBCL was made based on the International Consensus Classification of Mature Lymphoid Neoplasms: a report from the Clinical Advisory Committee and the 2022 revision of the World Health Organization classification of Haematolymphoid Tumours: Lymphoid Neoplasms (18, 19). The inclusion criteria were as follows (1): Age ≥ 18years (2), Tissue biopsy confirmed DLBCL, and the patient had not been treated before (3), No history of other major illnesses. The exclusion criteria for DLBCL patients were as follows (1): Patients with a history of other major diseases (e.g., mental illnesses, hepatitis or severe gastrointestinal diseases) (2), Patients who had used antibiotics within 30 days.

**Table 1:** Baseline data statistics of patients.

Features	ALL (n=87)	L-NCCN-IPI group (n=15)	LM-NCCN-IPI group (n=41)	MH-NCCN-IPI group (n=23)	H-NCCN-IPI group (n=8)
Gender					
Female	43	8	23	8	4
Male	44	7	18	15	4
**Age (years)**	60.59 ± 11.86	59.46 ± 11.31	60.04 ± 11.99	60.04 ± 12.04	60.46 ± 12.43
Ann Arbor					
I-II	41	13	24	4	–
III-IV	46	2	17	19	8
Extranodal involvement					
Yes	66	13	28	18	7
No	21	2	13	5	1
**LDH**	334.91 ± 256.81	305.64 ± 232.97	315.92 ± 238.72	321.68 ± 242.20	340.67 ± 257.50
ECOG performance status					
0-1	66	15	35	13	3
>1	21	–	6	10	5
**EBER**					
positive	12	1	5	4	2
negative	50	11	23	11	5
NA	25	3	13	8	1
Treatment					
R-CHOP based	87				
Response					
CR	58	13	31	13	1
PR	10	1	4	3	2
SD	2	1	0	1	0
PD	11	0	2	6	3
NA	6	0	4	0	2
Metagenomic sequencing					
Yes	69	14	33	16	6
No	18	1	8	7	2

ECOG, Eastern Cooperative Oncology Group; EBER, Epstein-Barr virus-encoded RNA; LDH, lactate dehydrogenase; L, low-risk; LM, low-intermediate-risk; MH, intermediate-high-risk; H, high-risk; NCCN-IPI, National Comprehensive Center Network-International Prognostic Indexes.

Both blood and feces samples were collected from naïve DLBCL patients before treatment. In detail, 5–10 mL of fresh peripheral blood was collected from 87 patients and stored at the EDTA containing tubes. A total of 69 qualified feces specimens were collected from 69 of the 87 patients, for which the inner middle section was intercepted with a fecal sampler and dispensed into 2 mL EP tubes with 0.5-2 g feces per tube.

Clinicopathological information was collected for each patient, including NCCN-IPI, age, Ann Arbor stage, extranodal involvement number, lactate dehydrogenase (LDH) level, and Eastern Cooperative Oncology Group (ECOG) performance status. Based on NCCN-IPI, the 87 patients were categorized into 4 groups, low-risk (L) group (0-1, n=15), low-intermediate-risk (LM) group (2-3, n=41), high-intermediate-risk (MH) group (4-5, n=23) and high-risk (H) group (6–8, n=8). Each participant provided the written informed consent. Our research was performed in accordance with the ethical standards formulated in the Helsinki Declaration and approved by the Ethics Committee of the First Affiliated Hospital of Zhejiang Chinese Medical University (2020-KL-027-02).

### 2.2 Metagenomic sequencing and data processing

Genomic DNA from 69 fecal samples was extracted using the EasyPure^®^ Stool Genomic DNA Kit (TransGen Biotech Co., Ltd, Beijing, China), and the metagenomic DNA library was constructed using the Nextera XT DNA Library Preparation Kit (Illumina, California, USA) following the manufacturer’s instructions. The quality of libraries was evaluated using an Agilent bioanalyser with a DNA LabChip 1000 Kit (Agilent, Palo Alto, USA). Samples with genomic DNA concentration less than 10 ng/μL, or abnormal quality (OD260/280>2.5, or OD260/280<1.5) were filtered out. Then, the cBOT was sequenced using the Illumina high-throughput sequencing platform NovaSeq6000 (Illumina, California, USA) according to the patent description provided by the Kangmeihuada Gene Technology Co., Ltd (Shenzhen, China) (20). After the low quality and ambiguous bases of the raw reads were filtered, the remaining reads were aligned to human genome reference (hg37) by KneadData to remove human host DNA contamination (21, 22). The average rate of host contamination was (1.53 ± 2.18) %.

### 2.3 Gene catalogue construction

Metagenomics of the Human Intestinal Tract (MetaHIT) gene catalogue was constructed according to previous descriptions (23). We performed *de novo* assembling and gene prediction for the high-quality reads of 69 samples using SOAPdenovo-28 (version 2.04). MetaGeneMark31 (version 3.26) was used to identify open reading frames (ORFs) from the contigs of each sample. Then, the non-redundant gene catalogue was constructed by pairwise comparison of all the predicted ORFs with CD-HIT32 (version 4.5.7). All predicted genes were aligned pairwise using BLAT. Genes that over 90% of their length can be aligned to another one with more than 95% identity (no gaps allowed) were removed as redundancies (24). MetaPhlAn (version 3.0) was used to perform species identification with default parameters. Microbiome constitution and abundance were analyzed using an absolute quantification method provided by the Kangmeihuada Gene Technology Co., Ltd (Shenzhen, China) (20).

### 2.4 Full-spectral flow cytometry

Full-spectral flow cytometry was used for immunophenotyping of B lymphocytes, T lymphocytes and NK lymphocytes of the peripheral blood samples (**Figure S1**). Detailly, the mixed non-bauble violet (BV) antibodies and brilliant staining buffer (Becton, Dickinson and Company, New York, USA) were added to the peripheral blood samples, and incubated for 15 min at room temperature in the dark. Then, lysis buffer (Becton, Dickinson and Company, New York, USA) was added to the samples, and the supernatant was discarded following 10 min of centrifugation. Sediment was washed with fixation/permeabilization buffer (Thermo Fisher Scientific, Massachusetts, USA) followed by centrifugation and incubated with cytoplasmic antibodies for 30 min in the dark. Phosphate buffered saline (Beyotime Biotechnology, Shanghai, China) was used to wash the samples. Finally, the supernatant was removed by centrifugation, and processed in a full-spectrum flow cytometer (Shanghai Xiatai Biotechnology Co., Ltd., Shanghai, China).

### 2.5 Statistical analysis

Sequencing data was analyzed using the corrplot (version 0.84), vegan (version 2.5-7) and ggplot2 packages of R (version 4.1.0). Phylogenetic tree was plotted by GraPhlAn (version 1.1.4) (25). Heatmap was drawn using the ComplexHeatmap (version 2.2.0) package in R (version 4.1.0) (26). Shannon index and Chao1 index were calculated using QIIME (27). Unifrac distance of principal component analysis (PCA) was generated using the scripts (Metaphlan, version 3.0) (28). PERMANOVA with the adonis function at 999 permutations was used from the vegan package. Dynamic Meta-Storms distance of principal coordinates analysis (PCoA) was generated using dynamic-meta-storms (version 1.1) tools (29).

The top20 most abundant microbes were plotted. RandomForest package (version 4.7-1) was used to identify species associated with NCCN-IPI scores. Differential abundance analysis was performed using Linear discriminant analysis effect size (LEfSe) (version 1.0.7), with the linear discriminant analysis (LDA) score >2 as a threshold for significantly different species. The bacterial abundance among different pathways were compared by Kruskal-Wallis test. Graphpad Prism (version 5.0) was used to plot the graphs and compare the differences of immune cell proportions and bacterial abundances between groups using Two-way ANOVA tests. *P* value <0.05 was considered statistically significant. * represents P < 0.05, ** represents P < 0.01, and *** represents P < 0.001.

The Simpson’s diversity index corresponding to each pathway was calculated. Pathways were excluded if they had non-zero abundance in DNA in <95% of the samples, or if >25% of the pathway was attributed to unclassified organisms (HUMAnN2) in >25% of the samples (30). The correlations between bacteria species and immune cell subtypes were tested using SPARCC with spearman method (5 inference iterations and 100 bootstrap). Absolute correlation value of ≥0.3 was considered as significant correlation.

## 3 Results

### 3.1 Microbial community composition in DLBCL patients and its correlation with clinical features

**Figure 1:**
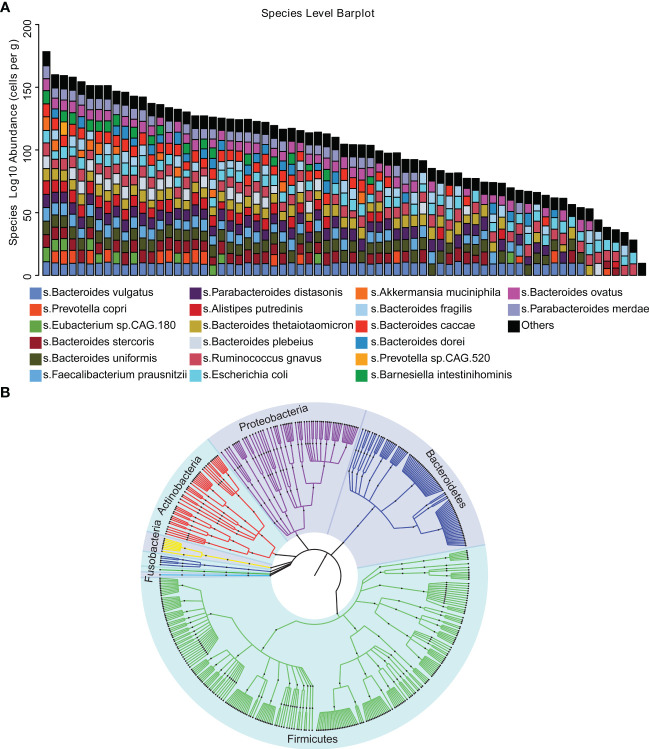
Microbial diversity mapping **(A)** Analysis of the gut microbiota in stool samples from 69 patients using absolute quantification. **(B)** Phylogenetic tree demonstrating that the five phylum bacteria of the gut microbiota exhibit continuous evolutionary relationships at six levels, including *Bacteroidetes*, *Firmicutes*, *Proteobacteria*, *Actinobacteria*, *Fusobacteria* and *Firmicutes*.

To investigate the gut microbiome characteristics of DLBCL, we performed metagenomic sequencing in 69 newly diagnosed DLBCL patients. A total of 462.48 Gb of high-quality PE reads were acquired with an average of 6.70 Gb per sample. In total, 10 bacterial phyla, 31 orders and 455 bacteria species were identified. The main bacteria species included *Bacteroides vulgatus*, *Prevotella copri*, *Eubacterium* sp.*CAG 180*, *Bacteroides stercoris*, *Bacteroides uniformis*, *Faecalibacterium prausnitzii*, *Parabacteroides distasonis*, *Alistipes putredinis*, *Bacteroides thetaiotaomicron*, *Bacteroides plebeius*, *Ruminococcus gnavus*, *Escherichia coli*, *Akkermansia muciniphila*, *Bacteroides fragilis*, *Bacteroides* caccae, *Bacteroides dorei*, *Prevotella* sp.*CAG 520*, *Barnesiella intestinihominis*, *Bacteroides ovatus* and *Parabacteroides merdae* (**Figure 1A**). The phylogenetic tree clearly demonstrated that there were 5 phyla of intestinal bacteria in 69 DLBCL patients, including *Bacteroidetes*, *Firmicutes*, *Proteobacteria*, *Actinobacteria* and *Fusobacteria*. *Firmicutes*, which showed a continuous evolutionary relationship in the biological classification (**Figure 1B**).

**Figure 2:**
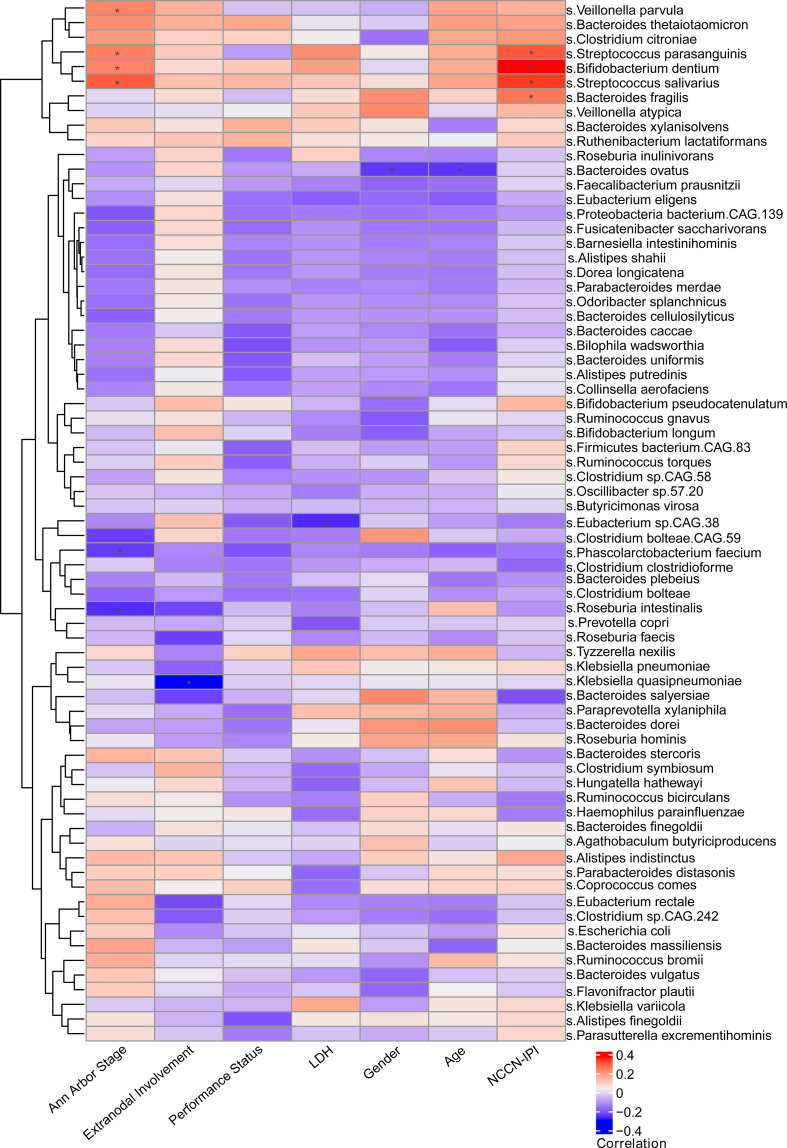
Relationship between clinical characteristics and gut microbiota Through heat map observation, it was found that there was a significant positive correlation between the clinical characteristics (Ann Arbor stage, NCCN-IPI) and gut microbiota in newly diagnosed DLBCL patients.

In addition, we assessed the association between intestinal bacteria constitution and the clinical features of patients with DLBCL, including NCCN-IPI, Ann Arbor stage, age, gender, LDH, extranodal involvement and ECOG performance status. The parallel heatmap demonstrated that *Roseburia intestinalis* and *Phascolarctobacterium faecium* were significantly negatively correlated with Ann Arbor stage, while *Streptococcus parasanguinis*, *Streptococcus salivarius*, *Veillonella parvula* and *Bifidobacterium dentium* were significantly positively correlated with Ann Arbor stage. Notably, *Streptococcus parasanguinis*, *Streptococcus salivarius*, *Bacteroides fragilis* and *Bifidobacterium dentium* were significantly positively correlated with NCCN-IPI (**Figure 2**). As NCCN-IPI is a prognostic index of DLBCL, we conjectured that the intestinal bacteria may be linked to the prognosis of DLBCL. In addition, there was no significant difference of clinical factors, such as gender, age, extranodal involvement and LDH, between different NCCN-IPI groups.

### 3.2 Diversity of intestinal bacteria in DLBCL patients with different NCCN-IPIs

**Figure 3:**
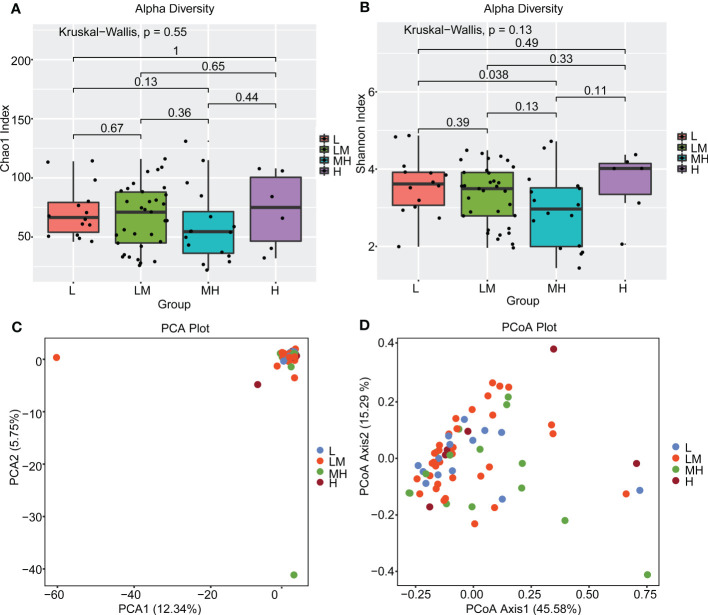
Diversity analysis of gut microbes between different NCCN-IPI groups The gut microbiota of the four groups of patients was analyzed using α diversity analysis based on **(A)** Chao1 and **(B)** Shannon index, respectively. β diversity among the four groups of patients was analyzed using **(C)** PCA and **(D)** PCoA.

To explore the association between intestinal bacteria and the prognosis of DLBCL, we further analyzed the association between intestinal bacteria and NCCN-IPI in patients with newly diagnosed DLBCL. First, we compared the diversity of intestinal bacteria in patients from the L, LM, MH and H groups using the alpha diversity (α-diversity) index. The MH group showed lower biodiversity according to the Chao1 and Shannon indices, but no significant difference was found between the four groups (**Figures 3A, B**). Also, the PCA and PCoA (β-diversity) were used to further analyze the differences in species diversity among different NCCN-IPI groups. The PCA results revealed no significant separation in bacterial community composition among the four groups using the first two principal component scores of PC1 and PC2, 12.34% and 5.75% of explained variance, respectively (**Figure 3C**). The PCoA results showed that the four groups of samples were close (**Figure 3D**). These results revealed a similar constitution of intestinal bacteria among different NCCN-IPI groups in DLBCL patients.

### 3.3 Different bacterial species identified in DLBCL patients with different NCCN-IPIs

**Figure 4:**
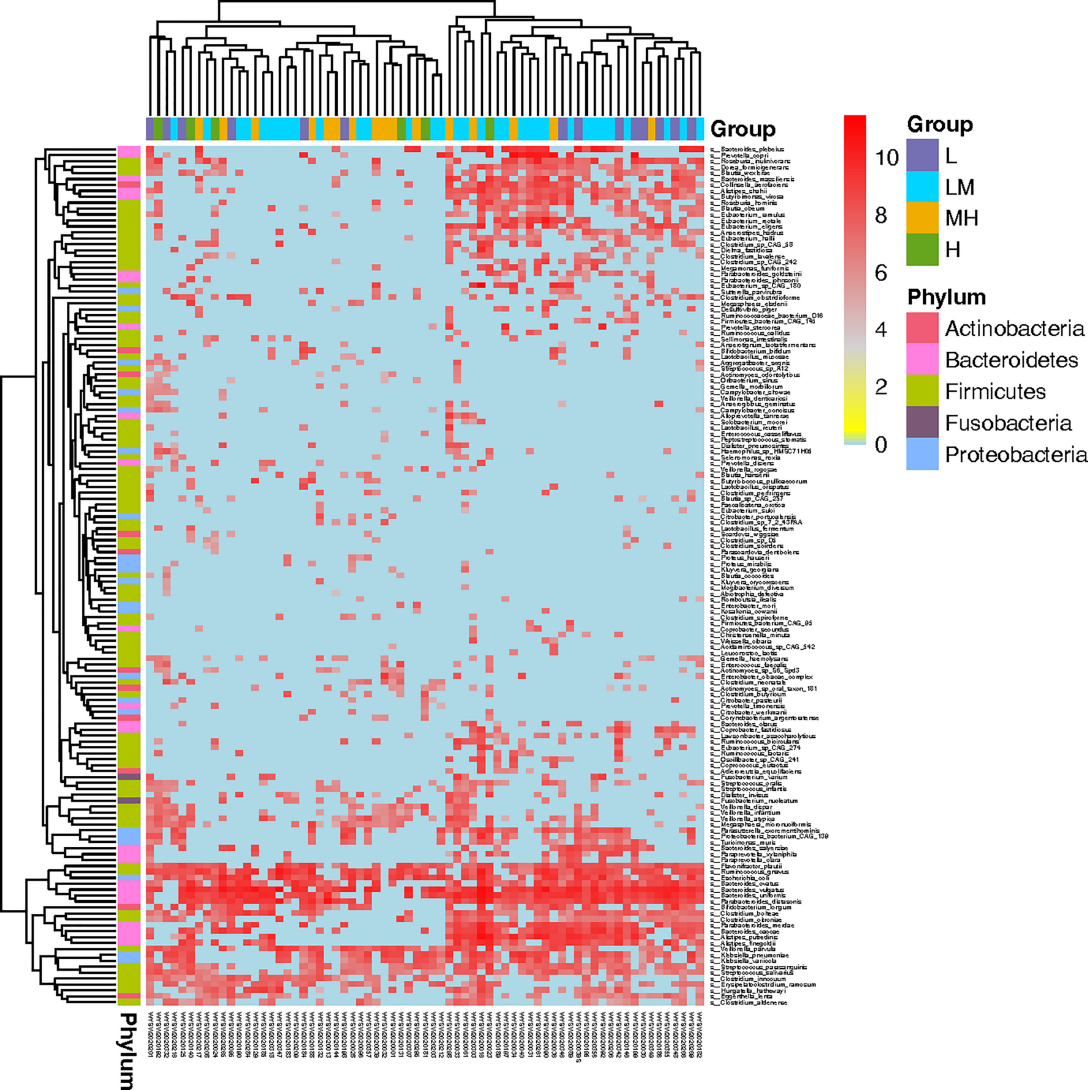
Screening of microbiota associated in DLBCL patients with different NCCN-IPIs The heatmap showed the Top145 differently accumulated bacterial species among DLBCL patients with different NCCN-IPIs.

Then, we screened the differently accumulated bacterial species among DLBCL patients with different NCCN-IPIs. The heatmap showed bacteria including *Bacteroides_plebeius*, *Prevotella_copri*, and *Roseburia_inulinivorans*, were mainly enriched in the L and LM groups (**Figure 4**).

**Figure 5:**
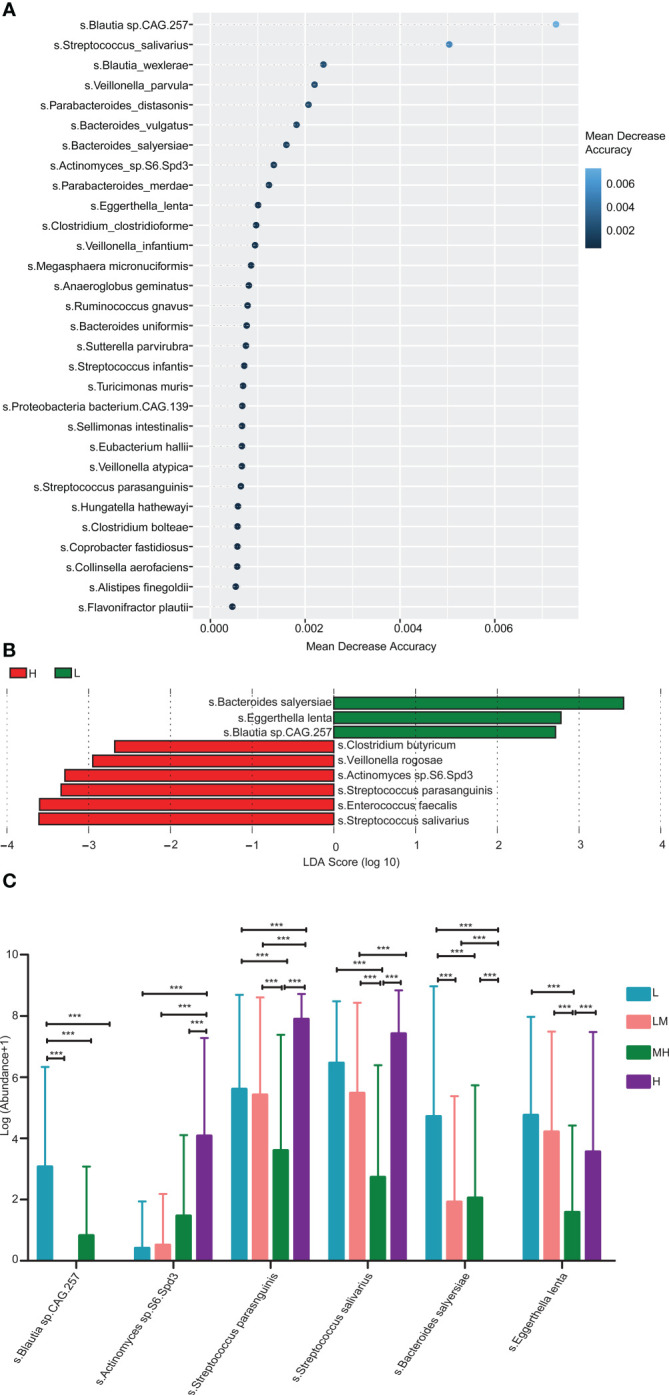
Enrichment pathway analysis of gut microbiota identified in 69 newly diagnosed DLBCL patients **(A)** The top 30 differential bacterial were identified by applying RandomForest regression of their relative abundances in DLBCL against NCCN-IPI. **(B)** LEfSe analysis was used to analyze the differences in microbiota between groups. **(C)** Six differential bacterial groups were screened according to both RandomForest and LEfSe analysis results.

In addition, we established a random-forest machine-learning model to further search the different bacterial species among different NCCN-IPI groups. Top30 bacterial species were demonstrated, such as *Blautia* sp.*CAG 257*, *Streptococcus salivarius*, *Blautia_wexlers, Veillonella_parvulas* and *Parabacteroides_distasonis* (**Figure 5A**). LEfSe analysis showed that 9 bacterial species with various abundances were found among the different NCCN-IPI groups. In detail, *Bacteroides salyersiae*, *Eggerthella lenta* and *Blautia* sp.*CAG 257* were significantly enriched in the L group, while *Clostridium butyricum*, *Veillonella rogosae*, *Actinomyces* sp.*S6 Spd3*, *Streptococcus parasanguinis*, *Enterococcus faecalls* and *Streptococcus salivarius* exhibited higher abundances in the H group (**Figure 5B**). Through integration the results of random-forest and LEfSe, 6 species of bacteria were identified, including *Blautia* sp.*CAG 257*, *Actinomyces* sp.*S6 Spd3*, *Streptococcus parasanguinis*, *Bacteroides salyersiae*, *Enterococcus faecalls* and *Streptococcus salivarius* (**Figure 5C**). Specifically, *Streptococcus parasanguinis* and *Streptococcus salivarius* were significantly accumulated in the H group (**Figure 5C**), while *Bacteroides salyersiae* was not detected in the H group (**Figure 5C**). Taken together, these results suggested that *Streptococcus parasanguis* and *Streptococcus salivarius* were accumulated in DLBCL patients with higher NCCN-IPI.

### 3.4 Enriched pathways of bacterial species identified in DLBCL patients

**Figure 6:**
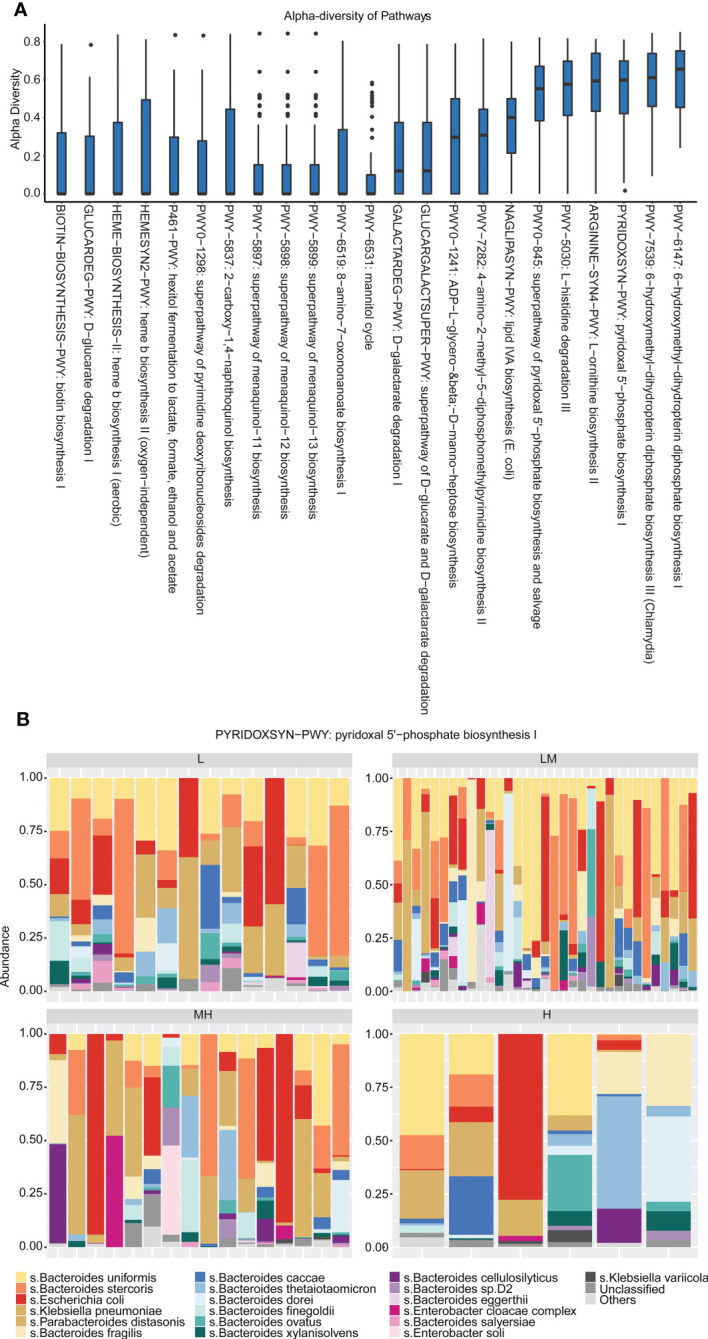
Enrichment pathways of differential gut microbiota **(A)** The relative abundance of microorganisms in each pathway was analyzed using Simpson’s diversity index. **(B)** The distribution of gut microbiota in each group in the Pyridoxal 5’-phosphate biosynthesis I pathway.

The Simpson’s diversity index was applied to analyze the enriched pathways of the 455 bacteria species discovered in the 69 newly diagnosed DLBCL patients. The results showed that the 455 bacteria species were mainly enriched in the Pyridoxal 5’-phosphate biosynthesis I pathway with a Simpson diversity index close to 1, indicating that bacteria species in this pathway had the greatest diversity (**Figure 6A**). Moreover, *Bacteroides uniformis*, *Bacteroides stercoris*, *Escherichia coli*, *Klebsiella pneumoniae* and *Parabacteroides distasonis* were involved in Pyridoxal 5’-phosphate biosynthesis I pathway in all of the four groups (**Figure 6B**). These findings suggested that bacteria species discovered in the newly diagnosed DLBCL patients were mainly enriched in the Pyridoxal 5’-phosphate biosynthesis I pathway.

### 3.5 Peripheral immune cell subtypes associate with NCCN-IPI in DLBCL

**Figure 7:**
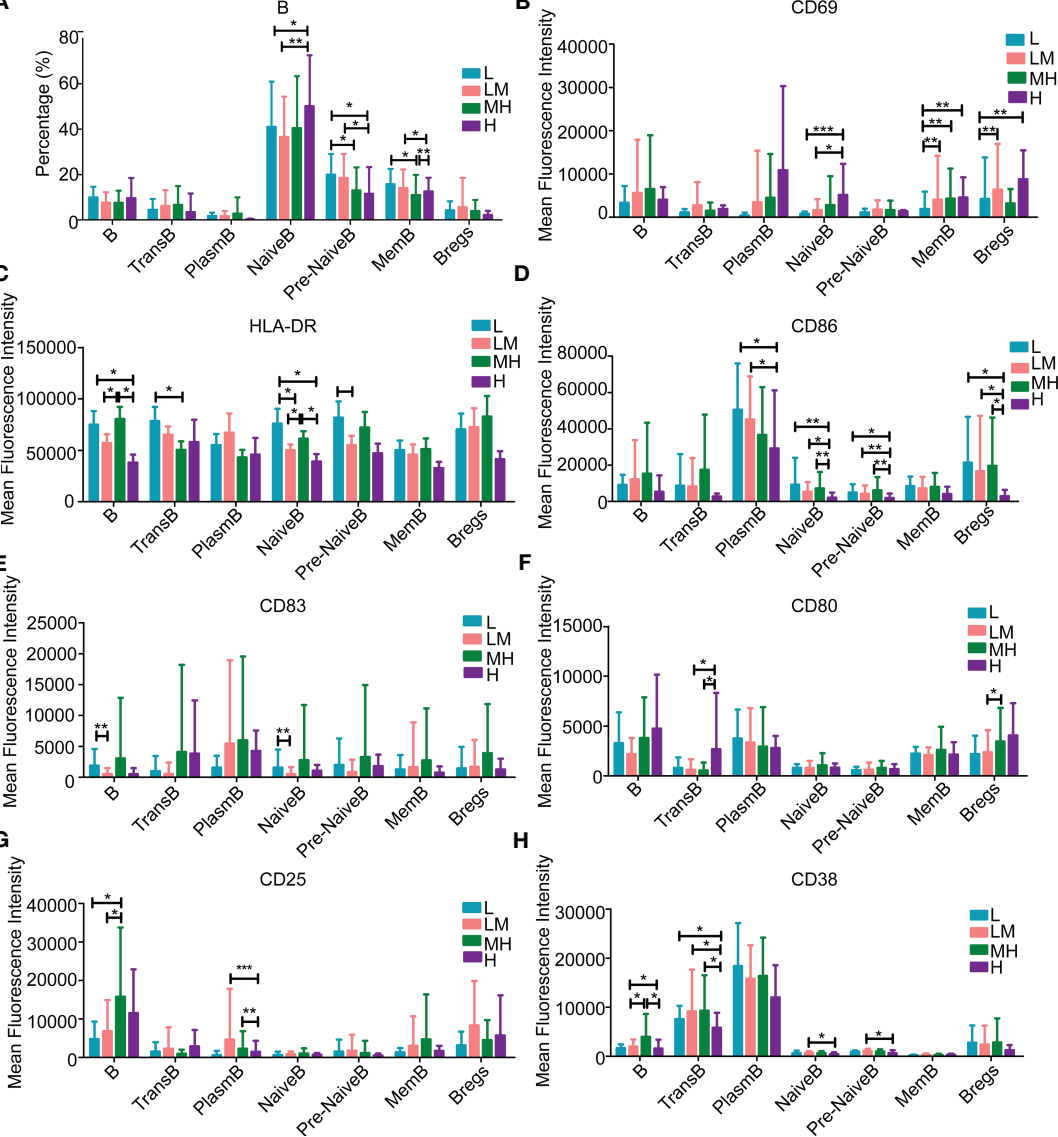
Populations of B lymphocytes in the peripheral blood samples of patients with different NCCN-IPIs The content of B lymphocytes **(A)** in each period. The mean fluorescence intensity of **(B)** CD69, **(C)** HLA-DR, **(D)** CD86, **(E)** CD83, **(F)** CD80, **(G)** CD25, **(H)** CD38 in each period of B lymphocytes. TransB: Transfer B cell; PlasmB: Plasmablast B cell; MemB: Memory B cell; Bregs: Regulatory B cell.

In addition, we explored the relationship between peripheral immune cell subtypes and NCCN-IPI in DLBCL patients. B lymphocytes, T lymphocytes and NK lymphocytes in the L, LM, MH and H groups were identified and quantified using Full-spectral flow cytometry. The screening process of T, B and NK lymphocytes was shown in **Figure S1**. The markers of immune cells were summarized in **Figure S2**. Among the B lymphocytes, H group showed a higher population of naïve B cells while a lower population of pre-naïve B cells as compared with other groups (**Figure 7A**). The populations of CD69+ naïve B cells, memory B cells and regulatory B cells were increased in H group (**Figure 7B**). A declined trend of HLA-DR+ B cells, transfer B cells and plasmablast B cells was observed in the H group (**Figure 7C**), as well as the CD86+ plasmablast B cells, naïve B cells, pre-naïve B cells and regulatory B cells (**Figure 7D**). Compared with LM group, L group showed a higher population of CD83+ B cells and naïve B cells (**Figure 7E**). In addition, an increased trend of CD80+ B cells, transfer B cells and plasmablast B cells was observed in the H group (**Figure 7F**), and the populations of CD25+ or CD38+ B cells were increased in MH group (**Figures 7G, H**).

**Figure 8:**
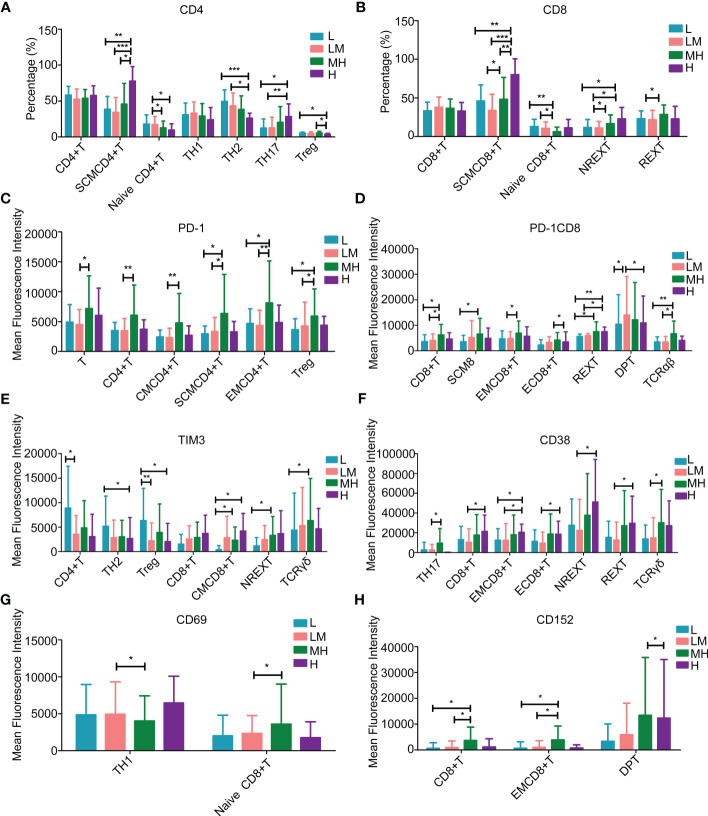
Populations of T lymphocytes in peripheral blood samples of patients with different NCCN-IPIs The content of **(A)** CD4 and **(B)** CD8 in T lymphocytes at each period. The mean fluorescence intensity of **(C)** PD-1, **(D)** PD-1CD8, **(E)** TIM3, **(F)** CD38, **(G)** CD69, **(H)** CD152 in each period of T lymphocytes. SCMCD4+ T: Stem cell-like memory CD4+T cell; SCMCD8+T: Stem cell-like memory CD8+ T cell; Treg: Regulatory T cell; REXT: Rescue exhausted T cells; NREXT: Unrescue exhausted T cells; EMCD4+ T: Effector memory CD4+T cells; EMCD8+ T: Effector memory CD8+ T cells; CMCD4+T: Central memory CD4+ T cell; CMCD8+ T: Central memory CD8+ T cell; DPT: CD4/CD8 double-positive T.

Among the T lymphocytes, the H group showed higher populations of stem cell-like memory (SCM) CD4+T cells and T-helper 17 (TH17) cells, while lower populations of T-helper 2 (TH2) cells and regulatory T (Treg) cells as compared with other groups (**Figure 8A**). The populations of SCM CD8+ T cells and nonrescue exhausted T (NREXT) cells were increased in H group (**Figure 8B**). Compared with other groups, HM group showed a higher population of all kinds of PD-1+ or PD-1+CD8+ T cells, except for CD4/CD8 double-positive (DP) T cells (**Figures 8C, D**). L group showed a higher population of TIM3+CD4+ T cells, TH2 cells and Treg cells while a lower population of TIM3+ central memory (CM) CD8+ T cells, NREXT cells and T cell receptor δγ+ (TCRδγ) cells as compared with other groups (**Figure 8E**). An increased trend of CD38+CD8+ T cells, NREXT cells and rescue exhausted T (REXT) cells were observed in H group (**Figure 8F**), as well as CD69+ T-helper 1 cells (**Figure 8G**). Compared with other group, HM group showed a higher population of CD152+CD8+ T cells, effector memory (EM) CD8+ T cells and CD4/CD8 DPT cells (**Figure 8H**).

**Figure 9:**
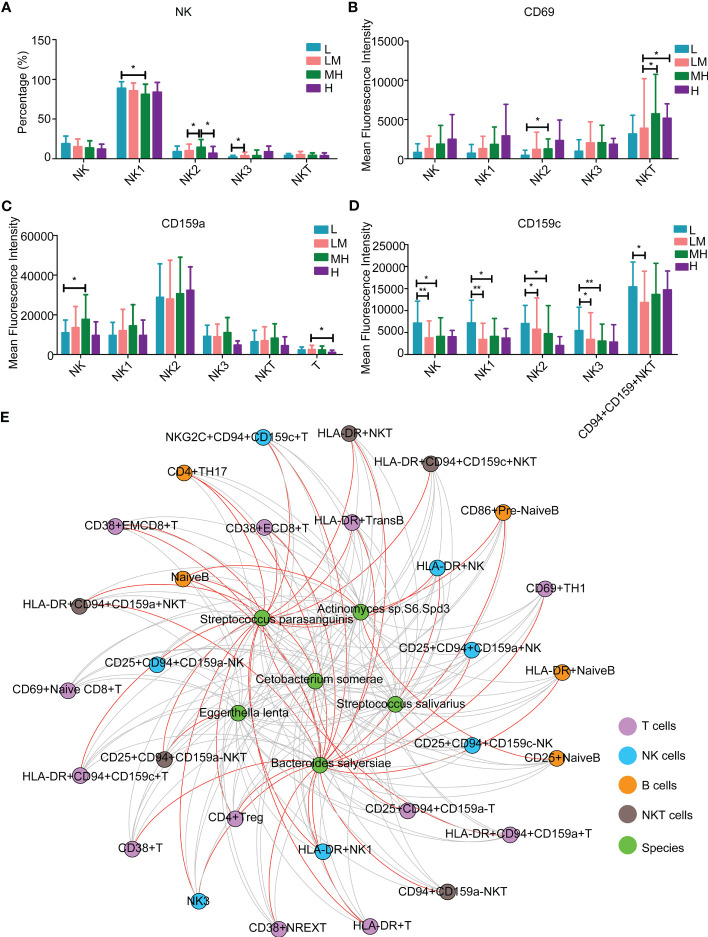
The relationship between peripheral immune cells and microbiotaThe content of NK lymphocytes **(A)** in each period. The mean fluorescence intensity of **(B)** CD69, **(C)** CD159a, **(D)** CD159c in each period of NK lymphocytes. **(E)** Interaction network diagram between 6 differential flora and differential immune markers.

Among the NK lymphocytes, MH group showed a higher population of NK2 cells while a lower population of NK1 cells as compared with other groups (**Figure 9A**). The populations of CD69+ NKT cells were increased in MH group (**Figure 9B**). Compared with the LM group, the L group showed a lower population of CD159a+ NK cells (**Figure 9C**) and an increased trend of CD159c+ NK/NK1/NK2/NK3/CD94+CD159+ NKT cells (**Figure 9D**). These results revealed various immune microenvironments between newly diagnosed DLBCL patients with different NCCN-IPIs.

### 3.6 Bacterial species interact with peripheral immune cell subtypes

Moreover, we investigated the association between the above mentioned 6 different bacteria species and 32 detected blood immune cell subpopulations. *Streptococcus parasanguinis* and *Bacteroides salyersiae* showed a stronger correlation with immune cells compared to other four bacteria species (**Figure 9E** and **Table 2**). In detail, *Bacteroides salyersiae* was mainly negatively associated with NK lymphocytes and T lymphocytes, including CD4+ Treg, CD38+ NREXT, NK3 and CD38+CD8+ EMT cells. *Streptococcus parasanguinis* was mainly negatively associated with NK lymphocytes and T lymphocytes, including HLA-DR+CD94+CD159c+ NKT, HLA-DR+ NK, HLA-DR+ NK1 and CD4+ Treg cells (**Table 2**). These results revealed a close association between intestinal bacteria and peripheral immune cells in DLBCL patients.

**Table 2:** Peripheral immune cells significantly associated with gut microbiota.

Gut	Peripheral immune cells	Cor	*P* value
Bacteroides salyersiae			
	Treg	-0.3360	0.0048
	CD38+ NREXT	-0.3298	0.0057
	NK3	-0.3283	0.0059
	CD38+EMCD8+ T	-0.3017	0.0118
Streptococcus parasanguinis			
	HLA-DR+CD94+CD159c+ NKT	-0.3466	0.0035
	HLA-DR+ NK	-0.3187	0.0076
	HLA-DR+ NK1	-0.3109	0.0093
	Treg	-0.3008	0.0120

Treg: Regulatory T; EMCD8+T: Effector memory CD8+T; NREXT: nonrescue exhausted T.

## 4 Discussion

In this study, we first described the pyridoxal of DLBCL and explored its association with immune microenvironment and NCCN-IPI, which was widely used for the prognosis assessment of patients with DLBCL (17). A total of 10 bacterial phyla, 31 orders and 455 species were identified in 69 patients with newly diagnosed DLBCL.

The diversity of bacteria showed no obvious difference between DLBCL patients with different NCCN-IPIs, which may be related to the perturbed gut microbe prior to treatment (31). Noticeably, we found the abundance of *Streptococcus parasanguinis* and *Streptococcus salivarius* were significantly increased in patients of the H group through integrating the association, Lefse and RandomForest results. *Streptococcus parasanguinis* is a dominant oral commensal and opportunistic pathogen for subacute endocarditis (32), and links to the prognosis of cancers. For instance, a significantly longer overall survival (OS) was observed in cutaneous melanoma patients carrying *Streptococcus parasanguinis* as compared to the non-carriers (33). *Streptococcus salivarius* consists of two clusters of strains with genomic and functional specificities (34). In 2018, Gavazza et al. (35) analyzed the gut microbiome of dogs with multicenter lymphoma and found that the abundance of *Streptococcus* was significantly higher in dogs with lymphoma as compared to that in the healthy dogs. In addition, *Streptococcus pneumoniae* predisposes to sepsis in patients with leukemia or lymphoma (36). Our study demonstrated for the first time that the abundance of *Streptococcus salivarius* was significantly elevated in patients with high NCCN-IPI, which may be a new direction for finding prognostic biomarkers for DLBCL patients.

Furthermore, we found that the bacteria species detected in the newly diagnosed DLBCL patients were enriched in the Pyridoxal 5’-phosphate biosynthesis I pathway, which had the highest diversity. Pyridoxal 5’-phosphate is the main component of vitamin B6, which is involved in the one-carbon metabolism of DLBCL, and thereafter affects the prognosis of DLBCL patients (37). Pyridoxal phosphates are produced by PDXK kinase from vitamin B6, and have been identified as an acute myeloid leukemia-selective dependency (38). Through inhibiting PDXK, the vitamin B6 pathway can be attenuated, thereby hindering the reproduction of leukemia cells (38). Thus, we hypothesize that intestinal microbiota may affect the progression of DLBCL through Pyridoxal 5’-phosphate biosynthesis I pathway, or more boldly vitamin B6, which needs to be verified in future studies.

In addition, our results revealed that the proportions of some kinds of immune cells were statistically different between different NCCN-IPI groups. For example, the populations of CD86+ plasmablasts cells, naïve B cells and pre-naïve B cells were decreased in DLBCL patients with higher NCCN-IPI. It has been reported that the loss expression of CD86, which was implicated in controlling tumor growth, was associated with decreased tumor-infiltrating T lymphocytes (T-TILs) in DLBCL patients (39). Besides, Xu et al. (40) found that DLBCL patients with a higher percentage of TIL-T always had a better survival. Taken together, these reports suggest that the high expression of CD86 predict a good outcome of DLBCL patients (39, 40). Consistently, our results showed that CD86+ B cells population was decreased in DLBCL patients with higher NCCN-IPI, suggesting that higher CD86+ might be a good prognosis marker for DLBCL. In addition, Dehghani et al. (41) demonstrated that higher peripheral IFN-γ/IL-4 T-helper 2 lymphocytes were associated with a favorable prognosis in DLBCL, which is consistent with our results that the proportion of T-helper 2 cells was higher in DLBCL patients with low NCCN-IPI. Moreover, we found that the proportion of CD8+ T cells was significantly increased in H group when compared with low NCCN-IPI, which was also reported in a previous report (42). In terms of TH17, it has been reported that TH17 cell number was increase DLBCL patients with high risk (43, 44). Similarly, our results showed that the percentage of TH17 cells was positively correlated with clinical stage, which suggested that increased TH17 cells may be associated with poor prognosis in DLBCL patients. These findings suggest that the immune cells were associated with the NCCN-IPI/prognosis of DLBCL patients, and may act as potential markers to evaluate the prognosis of DLBCL patients. In fact, many researchers have built prognostic evaluation models for DLBCL based on immune cells (42, 45, 46). Our results may provide further supporting clues for future relevant researches.

It has been proved that the gut microbiota is closely implicated in innate and adaptive immunity, and the clinical response to immunotherapy (47, 48). However, the association between gut microbe and immune cell subtypes in DLBCL patients has not been studied. *Streptococcus parasanguinis* was reported to be associated with the prognosis and immune checkpoint inhibitor therapy in cutaneous melanoma patients (33). *Bacteroides salyersiae* was involved in the regulation of immunotherapy resistance in renal cell carcinoma (49). However, their roles in regulating the curative effect of immunotherapy in DLBCL remain unclear. HLA-DR, a major histocompatibility complex class II, is mainly expressed in B lymphocytes, monocytes, macrophages, activated T lymphocytes, activated NK lymphocytes and progenitor cells (50). Low HLA-DR expression was associated with the poor prognosis of DLBCL patients receiving intensified upfront therapy (51). Higashi et al. (50) discovered that loss of HLA-DR expression in DLBCL led to a reduction of helper T-cells in the tumor microenvironment and was related to adverse outcome in DLBCL patients. Furthermore, it has been demonstrated that HLA-DR-positive DLBCL patients had longer OS when compared with HLA-DR-negative patients (52). Consistently, our results showed that HLA-DR+ cells showed a negative correlation with *Streptococcus parasanguinis*, which was more abundant in H group and was considered as a marker of poor prognosis in DLBCL, indicating HLA-DR is linked to good prognosis of DLBCL. The association between Tregs and DLBCL prognosis is controversial, some researchers found that the low number of Tregs was correlated with poor prognosis (53–55), while other researchers hold the opposite view or found no close relationship (56–58). Carreras J. et al. (59) found that low infiltration of FOXP3+ Tregs were associated with a poor prognosis in DLBCL. Similarly, in our study, we also discovered that the proportion of Tregs was decreased in the H group as compared with the L and MH group, suggesting that the higher Treg proportion may be associated with a better prognosis of DLBCL. In addition, our results showed that *Bacteroides salyersiae* was accumulated in the L group and *Streptococcus parasanguinis* was abundant in the H group. However, both of them were negatively correlated with the number of Tregs, suggesting a confusing role of Tregs in DLBCL patients.

## 5 Conclusion

This study reveals that *Streptococcus parasanguinis* and *Streptococcus salivarius* are enriched DLBCL patients with high NCCN-IPI score, and also show negative correlations with the proportions of peripheral immune cells (Tregs, CD38+ NREXT, NK3, HLA-DR+ NK, HLA-DR+ NKT, HLA-DR+CD94+CD159c+ NKT and CD38+EMCD8+ T cells). Our results indicate that the aberrant accumulation of *Streptococcus parasanguinis* and *Bacteroides salyersiae* may be the trigger of DLBCL and lead to peripheral immune system dysregulation. Collectively, this study first describes the gut microbiota landscape of DLBCL, and highlights the association between gut microbiota and NCCN-IPI, as well as the immune microenvironment, which may provide a new idea for the prognosis assessment and treatment of DLBCL.

## Data availability statement

The datasets generated for this study can be found in the National Genomics Data Center: https://ngdc.cncb.ac.cn/search/?dbId=&q=CRA009581.

## Ethics statement

The studies involving human participants were reviewed and approved by the Ethics Committee of the First Affiliated Hospital of Zhejiang Chinese Medical University. The patients/participants provided their written informed consent to participate in this study.

## Author contributions

Design of the work: JS, WQ, HY, YZ. Acquisition and analysis: YZ, SH, XX, LZ. Interpretation of data: YC, ZZ, XG, SZ, KY, LH, JF, YH, JJ. Drafted the manuscript: YZ. All authors contributed to the article and approved the submitted version.
