# Supplementary material for: Integration analysis of tumor metagenome and peripheral immunity data of diffuse large-B cell lymphoma
Source: Front Immunol. 2023 May 9;14:1146861. doi: 10.3389/fimmu.2023.1146861 (PMC10206395; doi:10.3389/fimmu.2023.1146861)
Supplement: Supplementary file 1 [file DataSheet_1.docx]

Supplementary Material

Integration analysis of tumor metagenome and peripheral immunity data of diffuse large-B cell lymphoma

**Yu Zhang^1^, Shuiyun Han^2^, Xibing Xiao^3^, Lu Zheng^4^, Yingying Chen^5^, Zhijian Zhang^6^, Xinfang Gao^7^, Shujuan Zhou^8^, Kang Yu^8^, Li Huang^7^, Jiaping Fu^6^, Yongwei Hong^5^, Jinhong Jiang^4^, Wenbin Qian^3*^, Haiyan Yang^2*^, Jianping Shen^1*^**

*** Correspondence:** Jianping Shen: [sjping88@163.com](mailto:sjping88@163.com); Wenbin Qian: [Qianwb@zju.edu.cn](mailto:Qianwb@zju.edu.cn); Haiyan Yang: haiyanyang1125@163.com.


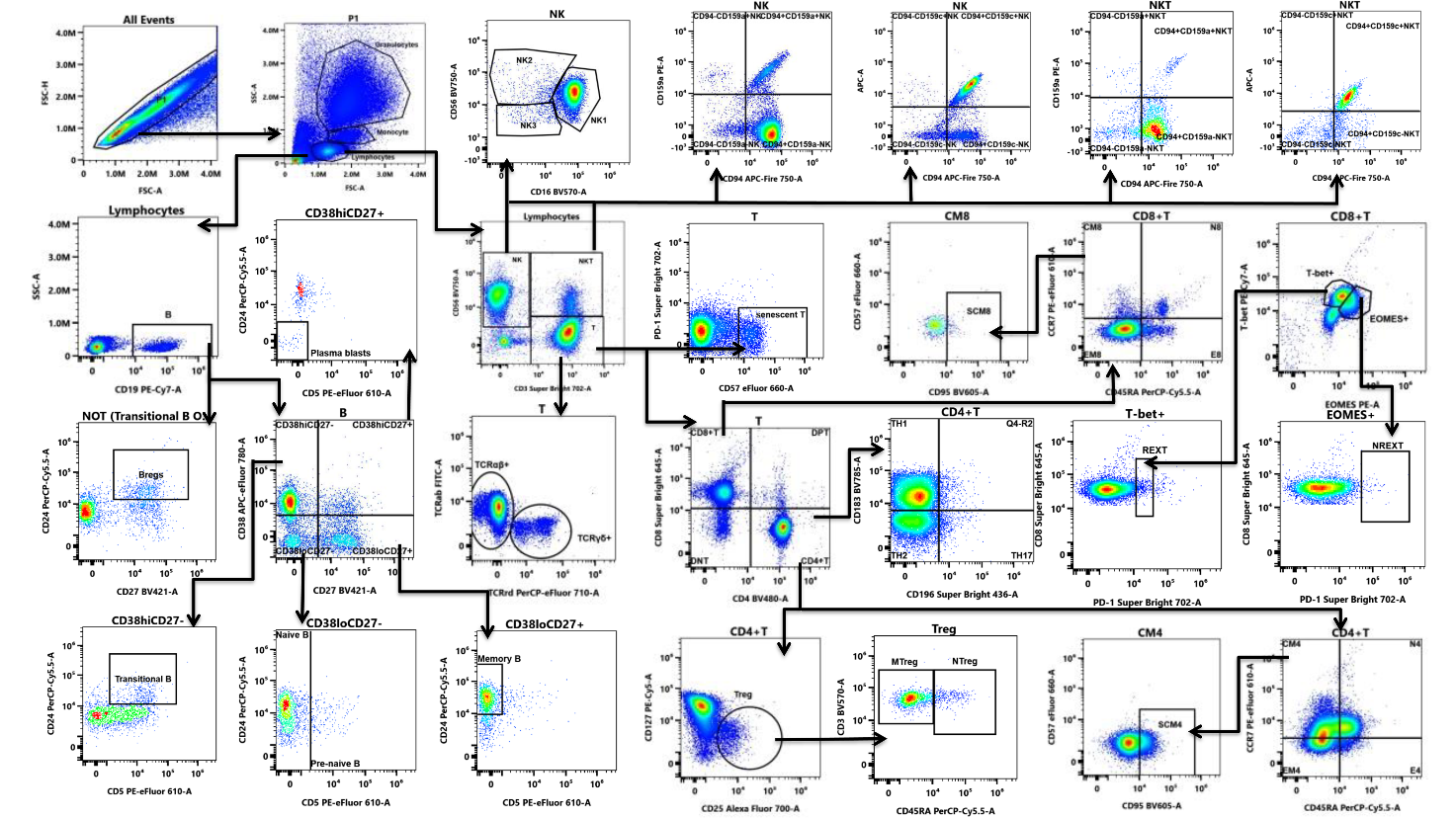
**Figure S1. Multicolor flow cytometry analysis of immune cell proportions in each group**

**
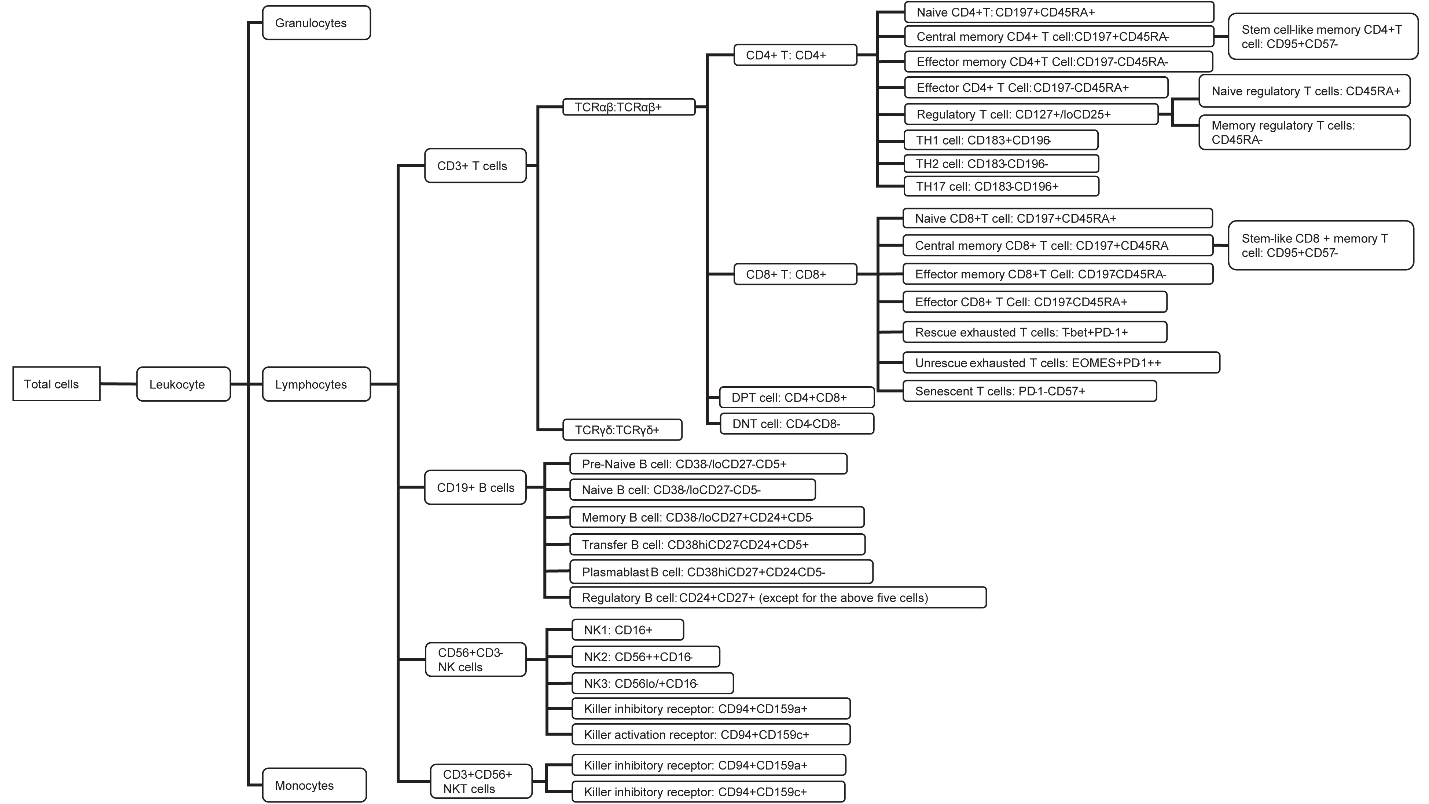
Figure S2. Types of immune cells analyzed in this study**
